# Scandium(III) Solvation and Association and Water Structure in the Gigapascal Pressure Range Investigated by Neutron Scattering

**DOI:** 10.3390/molecules30163417

**Published:** 2025-08-19

**Authors:** Toshio Yamaguchi, Sinichi Machida, Takanori Hattori

**Affiliations:** 1 Key Laboratory of Comprehensive and Highly Efficient Utilization of Salt Lake Resources, Key Laboratory of Salt Lake Resources Chemistry of Qinghai Province, Qinghai Institute of Salt Lakes, Chinese Academy of Sciences, Xining 810008, China; 2 Department of Chemistry, Faculty of Science, Fukuoka University, Jonan, Fukuoka 814-0180, Japan; 3 Neutron Science and Technology Center, Comprehensive Research Organization for Science and Society, Tokai, Naka 319-1106, Japan; s_machida@cross.or.jp; 4 J-PARC Center, Japan Atomic Energy Agency, Tokai, Naka 319-1195, Japan; takanori@post.j-parc.jp

**Keywords:** scandium, ion solvation, ion association, water, structure, neutron scattering

## Abstract

Scandium(III) (Sc(III)) is the smallest among the trivalent ions in Group 3, which includes yttrium(III) and lanthanides (III) with a hydration number of 8 and 8–9, respectively. The hydration number of Sc(III) in aqueous solutions reported so far varies from six to ten and remains an open question. In general, applying pressure and temperature to aqueous solutions perturbs the water structure and ion solvation, providing insight into the nature of ion solvation. In the present study, we perform neutron scattering measurements of a 1 *m* (mol/kg) ScCl_3_ aqueous solution in D_2_O (hereafter H is used to symbolize the hydrogen atom instead of D) under the thermodynamic conditions from 0.1 MPa/298 K to 4 GPa/523 K. Using the empirical potential structure refinement (EPSR) method, the neutron scattering data are analyzed to extract the site–site pair distribution functions, coordination number distributions, angle distributions, and spatial density functions (3D structure). A predominant Sc(III) species is [Sc(OH_2_)_7_]^3+^ with a distorted pentagonal bipyramidal geometry together with appreciable amounts of contact ion pair species [ScCl*_n_*(OH_2_)_(6−*n*)_]^(3−*n*)+^ (*n* = 1–3) and [Sc(OH_2_)_8_]^3+^ with mean Sc–Cl and Sc–OH_2_ distances of 2.42 and 2.11 Å, respectively. An aqua chloride ion is surrounded on average by 7.8 and 10.9 water molecules with a Cl–H_2_O distance of 3.10 Å at 0.1 MPa/298 K and 4 GPa/523 K, respectively. Applying GPa pressure transforms the tetrahedral network structure of water under ambient conditions to a dense, randomly packed structure with a mean coordination number of 12.6, resulting in an increase in the first-neighbor distance from 2.77 to 2.89 Å. The hydrogen bonds between water molecules remain linear but are largely distorted at high temperatures and high pressures. The present results provide a hint for understanding the underlying mechanism of high-pressure and temperature coordination chemistry and in applied fields, such as processes in geochemistry of the Earth’s upper mantle and pressure-induced protein denaturation.

## 1. Introduction

Scandium, Sc(III), is the first member of the trivalent ions in Group 3, followed by yttrium Y(III) and lanthanide Ln(III) (Ln = La to Lu). Sc(III) is more labile in ligand substitution reactions in aqueous solution than Y(III) [1]. Scandium is an important element from a variety of application fields, such as solvent extraction [2], scandia-stabilized zirconia for solid oxide fuel cells [3], and aluminum-scandium alloy of superior high strength and corrosion resistance [4]. For understanding the underlying mechanism in chemical processes and developing application technology, the structure of aqua Sc(III) is of great importance. The effective ionic radius of Sc(III) is 0.745 Å and 0.870 Å when the coordination number *CN* is 6 and 8, respectively, which is the smallest in the Group, in comparison with Y(III) (1.019 Å when the *CN* is 8) and Ln(III) (1.219 and 0.977 Å when the *CN* is 9 and 8, respectively), but larger than Al(III) (0.535 Å when the *CN* is 6) [5]. In aqueous solutions, it has been established that Al(III) is solvated by six water molecules, whereas the hydration number of Y(III) and La(III)–Lu(III) is 9 and 8, respectively [6]. Since Sc(III) is between Al(III) and Y(III) in size, the hydration number of Sc(III) is expected to be between six and eight. So far, various experimental methods, theoretical calculations, and their combined methods have been employed to reveal the solvation structure of Sc(III) in aqueous solution, including NMR [7], Raman scattering [8,9,10,11], large-angle X-ray scattering (LAXS) [12,13,14], extended X-ray absorption fine structure (EXAFS) [14,15], molecular dynamics (MD) simulation [15], quantum mechanical (QM) calculations [16,17], and a discrete variational Xα (DV-Xα) molecular orbital (MO) calculation [18]. Cotton reported a critical review of the Sc(III) aqua species in an aqueous solution and the crystalline states [19]. Table 1 summarizes the results obtained by these investigations. As seen in Table 1, the hydration number of Sc(III) is reported to be six, seven, eight, and ten, depending on the methods; the Sc(III)–Ow (Ow is the water oxygen atom) distance varies from 2.14 to 2.30 Å. In an aqueous solution containing a coordinating nitrate ion, Sc(III) forms an ion association with a nitrate ion [18]. Thus, the Sc(III) solvation structure in aqueous solution remains an open question.

In the crystal structures of ScX_3_.*n*H_2_O (X = Cl, Br) [20], the Sc(III) species has seven-coordination as [Sc(OH_2_)_7_]^3+^ in distorted pentagonal bipyramidal coordination geometry with Sc–Ow length of 2.09 Å in the axial direction and Sc–Ow length of 2.157–2.209 Å in the equatorial direction. In the crystalline [Sc(H_2_O)_8_](CF_3_SO_3_) compound [14], Sc(III) is coordinated by eight water molecules in a distorted bicapped trigonal prism geometry with six Sc–Ow bonds with 2.169 Å and two Sc–Ow bonds with 2.439 Å. In a crystal structure of [Sc(H_2_O)_6_][Sc(OSO_2_CH_3_)_6_] [11], the Sc(III) species has six-coordination environment expressed by [Sc(OH_2_)_6_]^3+^ in an octahedron with two Sc–Ow bonds with 2.085 Å, two bonds with 2.064 Å and two bonds with 2.092 Å. Thus, in the crystalline states, Sc(III) exhibits six-, seven-, and eight-water coordination, with mean Sc–Ow distances of 2.09, 2.16, and 2.19 Å, respectively, depending on the crystal field.

X-ray/Neutron scattering methods are a powerful tool for determining the detailed structure of ion solvation and association, as well as solvent water in aqueous electrolyte solutions. Neutron scattering has the advantage of locating light atoms, such as hydrogen, not available by X-ray scattering since neutrons are scattered by atomic nuclei whose scattering length of metal ions and hydrogen are comparable. It is needed, however, to use heavy water (D_2_O) as a solvent to avoid the incoherent scattering cross section of hydrogen (H), which is about 40 times larger than that of deuterium (D). Furthermore, empirical potential structure refinement (EPSR) modeling, combined with neutron scattering data, enables the extraction of site-site pair distribution functions, coordination number distributions, angle distributions, and spatial density functions (3D structure) of aqueous electrolyte solutions [21,22].

Applying pressure and temperature to aqueous solutions perturbs the tetrahedral network of solvent water structure (hydrogen bonding) [23,24], affecting the ion solvation and association [22]. Thus, the structure investigation of aqueous electrolyte solutions under high-pressure and high-temperature conditions is useful for elucidating the nature of ion solvation and association.

In the present study, we perform neutron scattering measurements on a 1 *m* ScCl_3_ aqueous solution under high-temperature and high-pressure conditions. Neutron scattering data are analyzed using EPSR to reveal the effects of pressure and temperature on the structure of Sc(III) solvation and association, as well as solvent water.

## 2. Results

### 2.1. Interference Functions and Pair Distribution Functions

**Figure 1:**
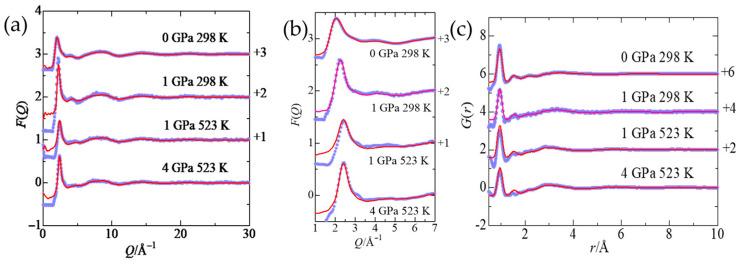
(**a**,**b**) The interference functions F(Q) and (**c**) the corresponding total pair distribution functions G(r) of a 1 *m* ScCl_3_ aqueous solution under various thermodynamic conditions. Blue filled circles: experimental, red solid lines: EPSR modeling.

Figure 1a,b, respectively, show the interference functions of a 1 *m* ScCl_3_ aqueous solution in the GPa pressure range. Figure 1c represents the corresponding total pair distribution functions (pdf). In Figure 1a,b, the first peak shifts from 2.05 to 2.20 Å^−1^ with increasing pressure to 1 GPa at 298 K. It further shifts to 2.45 Å^−1^ on heating to 523 K at 1 GPa. No more shift is observed upon compression to 4 GPa at 523 K. This phenomenon indicates the medium range order (i.e., intermolecular distances) are shortened by compression, as expected from the relation of d = 2π/Q; d is the distance and Q is the amplitude of the wavevector expressed as 4πsinθ/λ; 2θ is the scattering angle and λ is the neutron wavelength. Another pressure dependence is observed in the hump at Q = 4.05 Å^−1^ at 0 GPa/298 K (hereafter, 0.1 MPa is renamed as 0 GPa), which is a signature of the tetrahedral network structure of water molecules [23,24]. With increasing pressure and temperature, the hump gradually decreases and almost disappears at 4 GPa and 523 K [24] (Figure 1b). This result suggests that the tetrahedral network of solvent water is gradually broken down in the GPa pressure range and at 523 K. More quantitative discussion on the change in water structure in the GPa pressure range is given in Section 2.4. Figure 1c shows a well-defined peak at 0.95 Å and a small peak at 1.54 Å, attributed to the intramolecular O–D and D–D interactions within a water molecule, respectively. Pressure and temperature effects on the intramolecular structure of a water molecule were investigated by a least-squares fitting procedure applied to the interference functions in a Q-range of 10 to 30 Å^−1^, which are predominantly contributed from the intramolecular interactions of a water molecule. The details of a procedure are described in Supplemental Materials. Comparison between the experimental and fitted interference functions and the residual values is shown in Figure S1 (Supplementary Materials). The optimized structure parameter values are given in Table S1. The O–D distance of 0.959 Å obtained at 0 GPa/298 K in this study is somewhat shorter than the value (0.974 Å) reported by Kameda et al. [25]. The O–D distance within a water molecule in the liquid state is shorter than that (1.006 Å for hexagonal ice I_h_ [26] and 1.01 Å for cubic ice I_c_ [27]) in the crystalline state. Upon compression to the GPa pressure, the O–D distance is slightly lengthened to 0.963–0.970 Å, which is consistent with the values in high-pressure ice (0.961 Å for ice VI and 0.968 Å for ice VIII [28]).

### 2.2. Sc(III) Solvation

**Figure 2:**
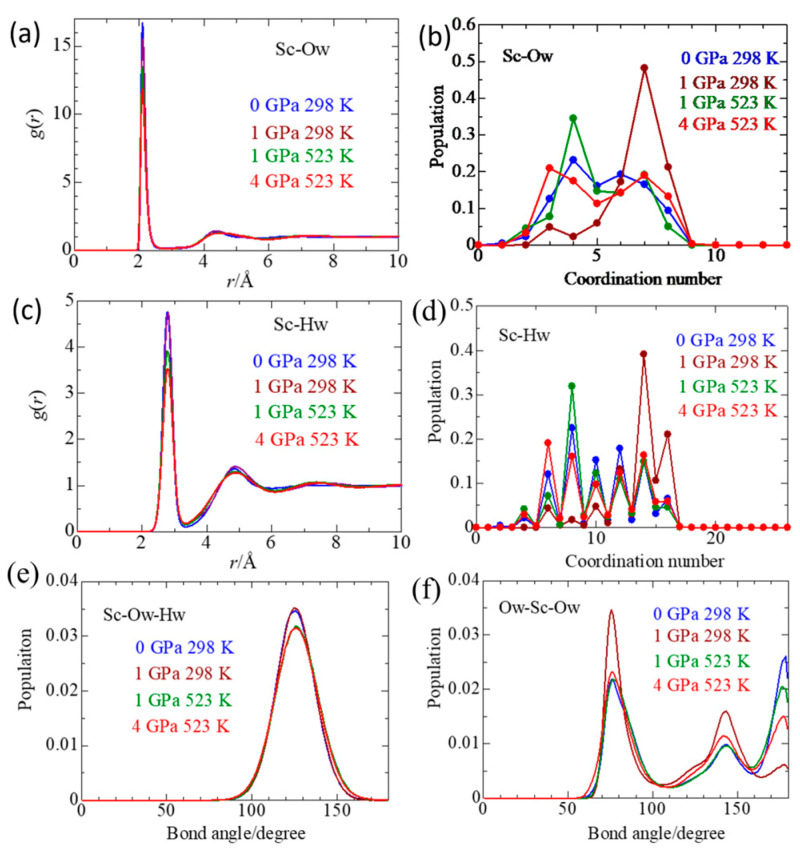
Pair distribution functions of Sc–Ow (**a**), Sc–Hw (**c**), and the corresponding coordination number distributions (**b**) and (**d**), respectively. (**e**) and (**f**) is the distribution of Sc–Ow–Hw and Ow–Sc–Ow angles, respectively, of 1 *m* ScCl_3_ aqueous solutions under various thermodynamic conditions.

**Figure 3:**
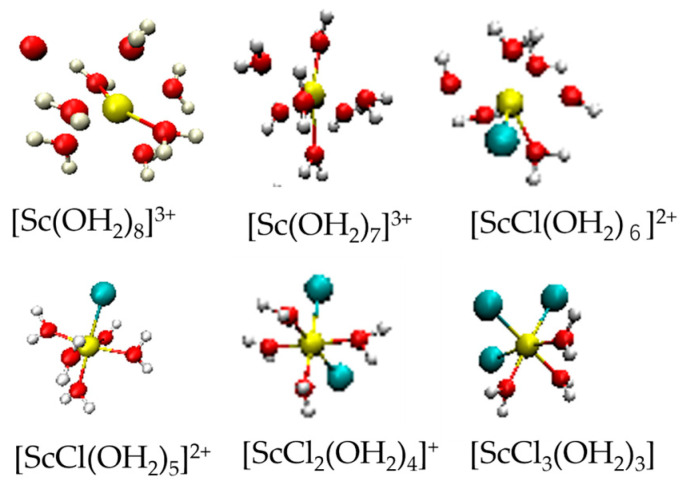
Snapshots of various Sc(III) species found from EPSR calculations of a 1 *m* ScCl_3_ aqueous solution in D_2_O under different thermodynamic conditions. Red, white, yellow, and blue balls represent the O, H, Sc, and Cl atoms, respectively.

Figure 2a shows the pdfs of the Sc–Ow pair in four different thermodynamic states. The positions of the peaks are given in Table 2. The first peak at 2.11 Å is ascribed to the Sc–Ow interactions in the first coordination shell. The present Sc–Ow distance is the shortest among those reported in the literature (Table 1). As pressure and temperature increase, the peak position remains unchanged, but the peak height gradually decreases. The water molecules in the second coordination shell of Sc(III) are located at 4.31 Å under ambient conditions and shift to a longer distance, 4.45 Å, at 4 GPa/523 K. Figure 2b shows the coordination number (CN) distributions of water molecules around Sc(III). The CN distribution of Sc–Ow at 0 GPa/298 K shows similar populations at CN = 4 (24%), 5 (17%), 6 (20%), and 7 (17%). When the pressure is applied to 1 GPa at 298 K, the CN population is the largest at CN = 7 (48%), CN = 6 (17%), and CN = 8 (21%). When the temperature is elevated to 523 K at 1 GPa, the population at CN = 7 decreases substantially to 19%, comparable with CN = 5 (16%), CN = 6 (15%), and CN = 7 (20%). At 4 GPa/523 K, the population at CN = 3 becomes 22%, comparable with CN = 4 (18%), CN = 5 (12%), CN = 6 (15%), CN = 7 (20%), and CN = 8 (14%). Figure 3 shows the snapshots of various Sc(III) species present in the simulation box of a 1 m ScCl_3_ aqueous solution in the four thermodynamic states. It is interesting to note that Sc(III) species of six-fold coordination are mono-, di-, and tricloro Sc(III) complexes. No aqua species [Sc(OH_2_)_6_]^3+^ was observed; instead, [ScCl(OH_2_)_6_]^2+^ was present, corresponding to CN = 6 for Sc–Ow pairs. The change in CN distribution with temperature and pressure can be explained by promoting the contact ion pair formation with increasing temperature. Table 2 shows the mean CN of water molecules of Sc(III). The mean CN is 6.6 ± 1.2 at 1 GPa/298 K. In contrast, those under the other thermodynamic states are 5.0–5.2, smaller by about one, which is compensated by a chloride ion (Cl^−^) entering the first coordination shell of Sc(III), as discussed in the next section.

Figure 2c shows the pdfs of Sc–Hw (Hw is the water hydrogen atom) at different pressures and temperatures. Figure 2d shows the coordination number distributions of Sc–Hw. The numerical values are summarized in Table 2. The first peak of Sc–Hw is observed at 2.71–2.81 Å. No temperature or pressure dependence was observed within the estimated experimental errors. The distribution of Sc–Hw coordination number amounts to double that of Sc–Ow, as expected. The peak height of Sc–Hw gradually decreases with increasing pressure and temperature.

Figure 2e,f show the distributions of Sc–Ow–Hw and Ow–Sc–Ow angles, respectively, under four thermodynamic conditions. From both figures, there are no significant changes in the orientational correlation of a bound water dipole and the coordination geometry of Sc(III) concerning temperature and pressure within the estimated experimental uncertainties. The Sc–Ow–Hw angle distribution has a maximum at 125°. Using the Sc–Ow and Sc–Hw distances and the intramolecular structure of a water molecule, we calculated the tilt angle of a water dipole between the Sc–Ow bond and a water dipole to be 0–25°. These results suggest that the water dipole orientation toward Sc(III) is not significantly perturbed by temperature and pressure and that CN = 6, 7, and 8 of the Sc(III) species remain preserved, independent of temperature and pressure, except for substitution between water molecules and chloride ions. The peaks of the Ow–Sc–Ow angle distribution are seen at 73°, 140°, and 180°. Since the predominant aqua species of Sc(III) is a seven-coordination, there are three possible coordination geometries: pentagonal bipyramidal (D_5h_), capped octahedral (C_2v_), and capped trigonal prismatic (C_2v_). Among these geometries, the pentagonal bipyramid is consistent with the feature of the Ow–Sc–Ow angle distributions. Figure 3 shows the snapshoots of various Sc(III) species obtained by EPSR modeling.

### 2.3. Sc(III) Association with Cl^−^

**Figure 4:**
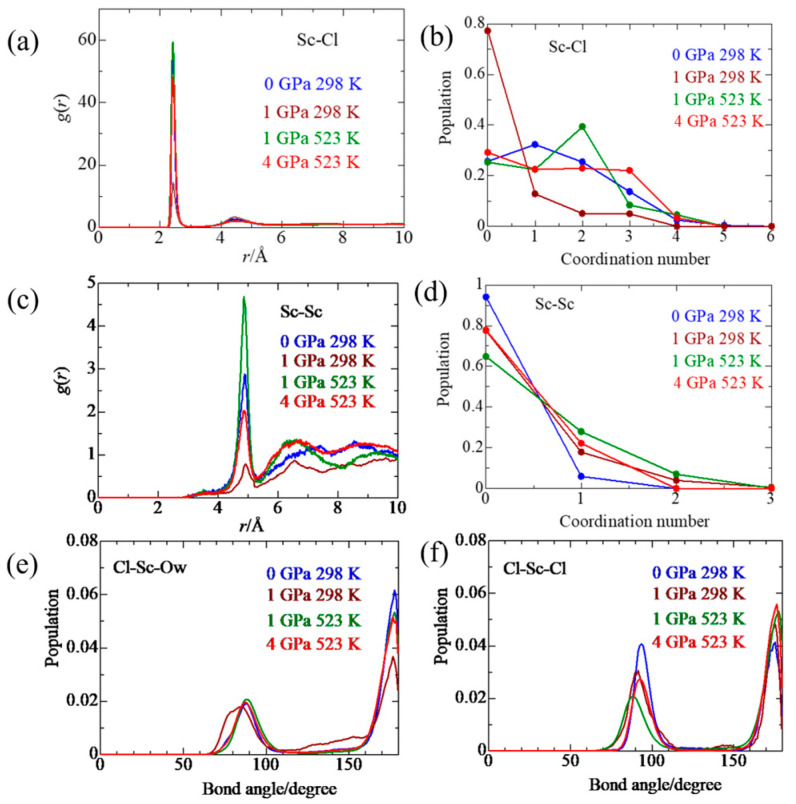
Pair distribution functions of Sc–Cl (**a**) and Sc–Sc (**c**), and the corresponding coordination number distributions (**b**,**d**), respectively. (**e**,**f**) show the distribution of Cl–Sc–Ow and Cl–Sc–Cl angles, respectively, of 1 *m* ScCl_3_ aqueous solutions under various thermodynamic conditions.

Figure 4a shows the pdfs of Sc–Cl in various thermodynamic states. The peak positions are given in Table 3. The first sharp peak at 2.42 Å corresponds to the contact Sc–Cl ion pairs since the distance agrees with a sum (2.415 Å) of ionic radii of Sc(III) (0.745 Å) and Cl^−^ (1.67 Å), whereas the second one at 4.44–4.53 Å is ascribed to the solvent (water) shared ion pairs, where a water molecule is shared with Sc(III) and Cl^−^. Figure 4b shows the CN distributions. At 0 GPa/298 K, the population of CN = 1 (33%) is the highest, followed by CN = 2 (26%), CN = 0 (26%), and CN = 3 (14%). When the pressure is applied at 298 K, the CN population shifts to CN = 0 (77%), CN = 1 (13%), and CN = 2 (5%) and CN = 3 (5%). With elevating temperature to 523 K at 1 GPa, the highest population is CN = 2 (41%), with CN = 0 (26%), CN = 1 (23%), and CN = 2 (8%). With further increasing pressure to 4 GPa at 523 K, the Sn(III) species of CN = 3 (23%) is newly formed together with CN = 0 (30%), CN = 1 (23%), and CN = 2 (24%). These results show that the equilibria of the contact ion pairs shift to the formation of the higher Sc(III) chloro complexes with increasing temperature and pressure. It is noted that applying pressure at 298 K suppresses the formation of the contact ion pairs. The mean coordination number of Sc–Cl pairs is a similar value of 1.4–1.5, except for 0.38 at 1 GPa/298 K; however, it should be noted that elevating temperature promotes the formation of higher Sc(III) chloro complexes.

Figure 4c,d show the pdfs of the Sc–Sc pair related to possible triple ion pairs Sc–Cl–Sc and the corresponding CN distributions, respectively. The distribution of CN = 1 is 6%, 19%, 30%, and 22% at 0 GPa/298 K, 1 GPa/298 K, 1 GPa/523 K, and 4 GPa/523 K, respectively. The mean CN of Sc–Sc is given in Table 3. At 298 K, the mean CN = 0.056 ± 0.24 suggests that there are no appreciable triplets, irrespective of pressure. When the temperature is raised to 523 K, the mean CN is increased to 0.43 ± 0.63 at 1 GPa and 0.22 ± 0.42 at 4 GPa, corresponding to the amount of triplets is 40% and 22%, respectively. The largest amount of the triplet at 1 GPa/523 K is due to the lowest dielectric constant, as discussed in Section 3.

Figure 4e,f show the distributions of Cl–Sc–Ow and Cl–Sc–Cl angles. At all thermodynamic states investigated, the angle distributions are dominant at 90° and 180°. As shown in Figure 3, the Sc(III) chloro complexes have an octahedral geometry, leading to Cl–Sc–Ow and Cl–Sc–Cl angles of 90° and 180°. Among the Sc(III) chloro complexes, the Sc(III) chloro complexes with two or three Cl occupying the trans conformation were not observed in the snapshots, but will probably be seen in other snapshots.

### 2.4. Chloride Ion Solvation

**Figure 5:**
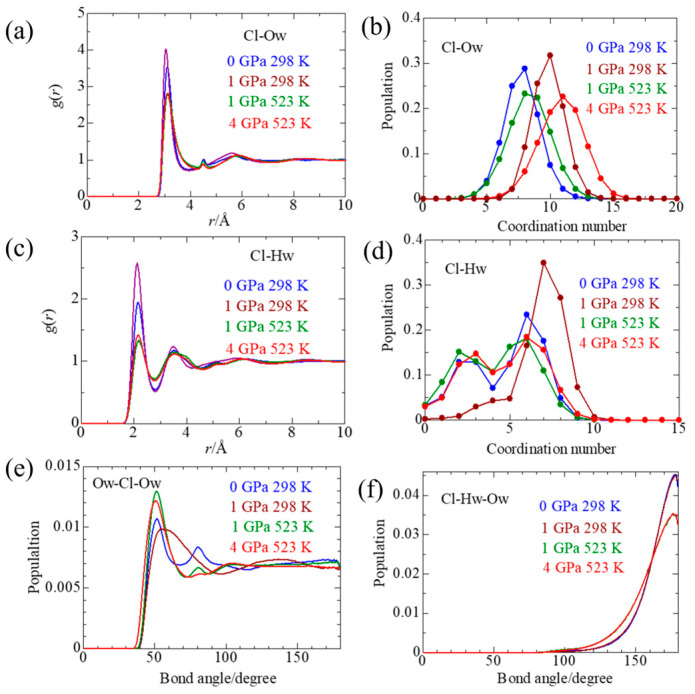
Pair distribution functions of Cl–Ow (**a**) and Cl–Hw (**b**), and the corresponding coordination number distributions (**c**,**d**), respectively. (**e**,**f**) is the distributions of Ow–Cl–Ow and Cl–Hw–Ow angles, respectively, of 1 *m* ScCl_3_ aqueous solutions under various thermodynamic conditions.

Figure 5a shows the pdfs of Cl–Ow pairs, respectively. The corresponding CN distributions are presented in Figure 5b. The numerical values of the peak positions and the mean CN are summarized in Table 2. The first peak position of the Cl–Ow pdf is 3.10 Å, irrespective of pressure and temperature. The present Cl–Ow distance is shorter than that (3.21 Å) previously reported [22]. The differences in bond lengths might be due to the potential parameter values of Cl^−^ employed. With increasing pressure and temperature, the peak height gradually decreases, and the peak shape becomes asymmetric toward the long-distance side. This feature suggests that water molecules outside the first solvation shell tend to approach Cl^−^ in a wider distance range of the solvation shell. The CN distribution has a peak at CN = 8 at 0 GPa/298 K, which shifts to CN = 10 at 1 GPa/298 K. When the temperature is elevated to 523 K at 1 GPa, the CN peak comes back to 8 and 9, but again shifts to 11 upon compression to 4 GPa at 523 K. Thus, applying pressure tends to break the solvation shell of Cl^−^.

A more detailed hydrogen-bonded feature of water molecules around Cl^−^ is observed in the pdfs of Cl–Hw in Figure 5c. The peak position of Cl–Hw(I) pdf is 2.12–2.14 Å at 298 K and shifts to 2.17–2.20 Å at 523 K. Figure 5d shows the CN distributions of Cl–Hw. The CN distributions of Cl–Hw show a similar feature: a main peak at CN = 6 and a subpeak at CN = 2–3, irrespective of pressure and temperature, except for a predominant peak at CN = 7 at 1 GPa/298 K, which is in marked contrast with that of Cl–Ow. Since Cl^−^ is associated with Sc(III) in the first solvation shell of Cl^−^, as discussed in the previous section, the peak at CN = 2–3 is ascribed to water hydrogen atoms bonded to the Cl^−^ bound to Sc(III). The peak at CN = 6 should be related to the Cl^−^ solvation. It should be noted that the CN distribution of Cl–Hw is shifted to a lower CN than that of Cl–Ow. This result suggests that non-hydrogen-bonded water molecules enter the solvation shell of Cl^−^ upon compression. The mean CN of Cl–Hw remains constant at 4.2–4.7, irrespective of pressure and temperature, which is in contrast with that of Cl–Ow, which increases with pressure.

Figure 5e,f show the Ow–Cl–Ow and Cl–Hw–Ow angle distributions, respectively. In the Ow–Cl–Ow angle distributions under ambient conditions (0 GPa/298 K), two peaks are observed at 50° and 80°. With increasing pressure and temperature, the peak at 80° gradually decreases and almost disappears at 523 K. At 4 GPa and 523 K, only a peak appears at 50°. In the GPa pressure range, only a major peak is observed at 50°. The mean CN is 9.7–10.9. As discussed in the next section, solvent water molecules take a densely, randomly packed structure, and chloride ions are encapsulated in it. The distributions of Cl–Hw–Ow angle (Figure 5f) provide a feature of the Cl–H_2_O hydrogen bonding. Only a sharp peak is observed at 180°, corresponding to a linear Cl–H–Ow bond. In the GPa pressure range, this linear hydron bond maintains, but the peak height decreases, and the peak becomes broader. These results suggest compression causes the Cl–H–Ow bond to be largely distorted.

### 2.5. Solvent Water

**Figure 6:**
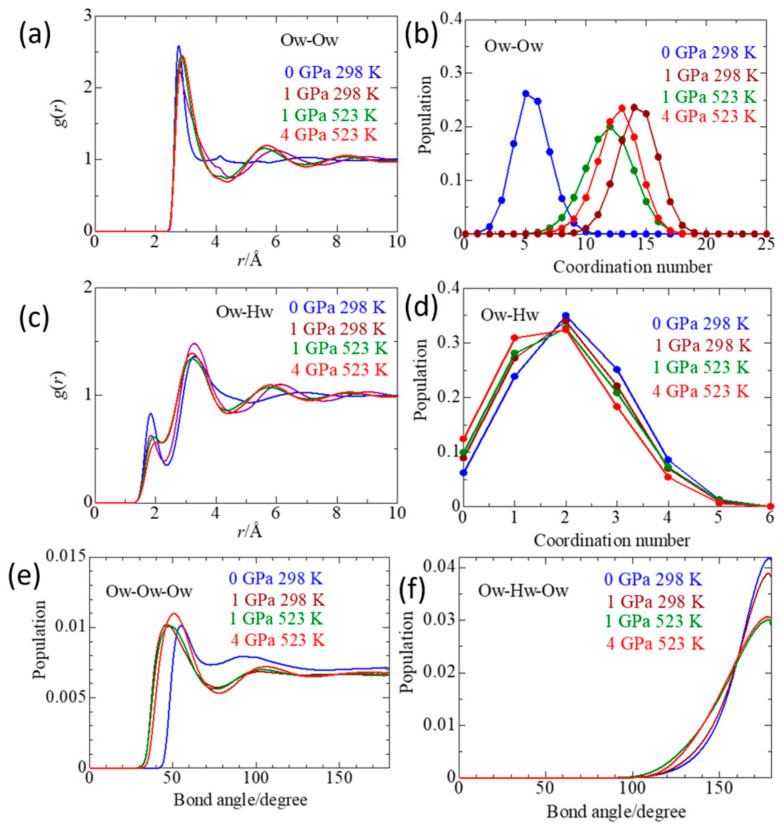
Pair distribution functions of Ow–Ow (**a**) and Ow–Hw (**c**), and the corresponding coordination number distributions (**b**,**d**), respectively. (**e**,**f**) is the distribution of Ow–Ow–Ow and Ow–Hw–Ow angles, respectively, of a 1 *m* ScCl_3_ aqueous solution under various thermodynamic conditions.

Figure 6a shows the pdfs of Ow–Ow pairs of solvent water, respectively. The peak positions of the pdfs are given in Table 4. The first peak position of Ow–Ow (I) is 2.77 Å at 0 GPa/298 K. The second Ow–Ow (II) and third Ow–Ow (III) peaks are observed at 4.13 and 7.12 Å, respectively. This feature is consistent with the tetrahedral network structure of water [23,24]. Upon compression to 1 GPa at 298 K, the first peak position does not change significantly. However, the peak becomes asymmetric to the longer distance side, indicating water molecules occupying the interstitial sites. Furthermore, the positions of the second and third peaks drastically change to 5.9 and 9.0 Å, respectively. This result suggests that the tetrahedral network structure of water is broken down at 1 GPa/298 K. With elevating temperature and further increasing pressure, the first peak is lengthened to 2.84–2.89 Å. The second and third peaks do not show further change in positions, probably due to the breakdown of the water structure at 1 GPa/298 K. Figure 6b shows the CN distribution of Ow–Ow.

At 0 GPa/298 K, the peak of the distribution is centered at five due to the tetrahedral network structure. When the pressure is increased to 1 GPa and 298 K, the CN distribution drastically moves to the one centered at CN = 14. With the temperature elevated to 523 K at 1 GPa, the peak of the CN distribution shifts back to 12. However, when the pressure is applied to 4 GPa, the peak of the CN distribution shifts again to 13. The mean CN changes from 5.5 at 0 GPa/298 K to 12.6–14.3 at in the GPa pressure range. The value of 12.6–14.3 is close to CN = 12 found for simple liquids like Ar [29], taking a dense random packing structure. Thus, the structure of water in the GPa pressure range is similar to a dense random packing structure.

Next, we see the details of the hydrogen bonds among water molecules in the pdfs of Ow–Hw shown in Figure 6c. The peak positions are given in Table 4. As seen in Figure 5c, the hydrogen bonding Ow–Hw(I) peak is observed at 1.83 Å at 0 GPa/298 K. With increasing pressure and temperature, the peak position gradually shifts to a longer distance: 1.86 Å at 1 GPa/298 K, 1.95 Å at 1 GPa/523 K, and 2.08 Å at 4 GPa/523 K. Furthermore, the peak height is gradually lowered, and the first minimum at 2.30 Å becomes shallower. These results show the partial breaking and/or distortion of the intermolecular hydrogen bonds between water molecules with pressure and temperature. The temperature and pressure dependence of the second Ow–Hw(II) and third Ow–Hw(III) peaks show a characteristic change in distance. The Ow–Hw(II) distance is gradually shortened from 3.28 Å at 0 GPa/209 K to 3.20 Å at 4 GPa/298 K, whereas the Ow–Hw(III) distance drastically decreases from 6.90 Å to 5.78 Å with pressure and temperature. These changes originate from a structural change from the tetrahedral network to a dense random packing.

Figure 6d shows the CN distribution of Ow–Hw, which does not change significantly with pressure and temperature, in marked contrast with that of Ow–Hw. The peak of the CN distribution remains centered at two in the four thermodynamic states, but the distribution of CN = 3 gradually decreases, whereas that of CN = 1 evolves. The mean CN of Ow–Hw gradually decreases from 2.1 ± 1.1 at 0 GPa/298 K to 1.8 ± 1.1 at 4 GPa/298 K. Thus, we can say that the intermolecular Ow–Hw is rather distorted than broken down with increasing pressure and temperature.

The hydrogen bonding feature between water molecules is further investigated concerning the Ow–Ow–Ow and Ow–Hw–Ow angle distributions, as seen in Figure 6e,f, respectively. Figure 6e shows an intense peak at 55° and a broad peak at 95° at 0 GPa/298 K. The latter peak corresponds to water molecules within the tetrahedral network structure, whereas the former corresponds to interstitial water molecules within the hydrogen bond network [23,24]. With increasing pressure to 1 GPa, the 95° peak disappears, and the main peak shifts toward a lower angle, corresponding to the distortion of the hydrogen bonds. The distribution of ∠Ow–Hw–Ow in Figure 5f shows a peak at 180°, characteristic of a linear hydrogen bond. When the pressure is increased to 1 GPa at 298 K, the peak slightly decreases. When the temperature is raised to 523 K, the peak substantially decreases and broadens, indicating the distortion of the Ow–Hw–Ow hydrogen bonds. It should be noted that the pressure effect on the Ow–Ow pairs is much larger than the Ow–Hw ones.

**Figure 7:**
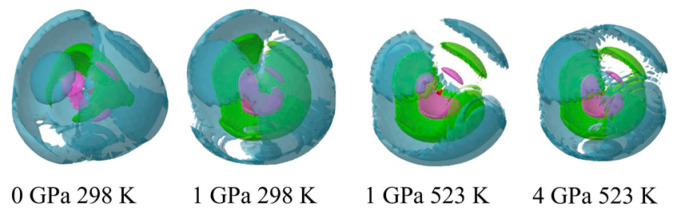
Spatial density functions of the water oxygen atoms in the first-neighbor (pink), second-neighbor (green), and third-neighbor (grey) shells around a central water molecule, shown with an oxygen atom (red ball) and hydrogen atoms (white balls) of solvent water in a 1 *m* ScCl_3_ aqueous solution under various thermodynamic conditions. The contour level is 0.15.

Figure 7 shows the SDFs of the water oxygen atoms in the first-neighbor, second-neighbor, and third-neighbor shells around a central water molecule under the four different thermodynamic conditions. Under ambient conditions, the tetrahedral network structure of water is seen. Upon compression to 1 GPa at 298 K, the second neighbor lobes are very broadened, and the tetrahedral network structure is largely distorted. When the temperature is elevated to 523 K at 1 GPa, and the pressure is raised to 4 GPa at 523 K, the tetrahedral network structure is no longer present, resulting in a densely random-packed structure. However, it should be noted that the tetrahedral moiety in the first shell of water maintains a large distortion.

## 3. Discussion and Conclusions

Previous studies on aqua Sc(III) species in aqueous systems have reported six-, seven-, eight-, and ten-coordinate water arrangements, depending on the methods employed. According to the present neutron scattering results, [Sc(OH_2_)_7_]^3+^ is predominantly formed in equilibrium with [Sc(OH_2_)_8_]^3+^ and Sc(III) chloro complexes, [ScCl(OH_2_)_6_]^2+^ and [ScCl*_n_*(OH_2_)_6−*n*_]^(3−*n*) +^ (*n* = 1, 2, and 3) in a 1 *m* ScCl_3_ aqueous solution. If the solution is diluted to less than 1 *m*, the formation of Sc(III) chloro complexes would be suppressed, owing to decreasing electrostatic interactions between ions. The aqua Sc(III) species [Sc(OH_2_)_6_]^3+^ would be formed as well as [Sc(OH_2_)_7_]^3+^ and [Sc(OH_2_)_8_]^3+^. In the crystalline state, the hexaaqua, heptaaqua, and octaaqua Sc(III) species are present as a single species, depending on their crystal fields. This fact shows that Sc(III) has a unique property of coordinating six to eight water molecules due to its smaller ionic radius, in contrast to those of Y(III) and Ln(III). In an aqueous solution, water molecules move around Sc(III) instantaneously without the influence of crystal fields in the solid state. Thus, it is plausible that Sc(III) takes three different species in an aqueous solution. In previous LAXS, EXAFS, and MD studies, the authors reported the mean coordination number and the running coordination number obtained using Equation (4). LAXS data contain all the site-site pdfs. They are thus difficult to use to uniquely determine the coordination number of Sc(III) and water interactions. EXAFS at the Sc *K*-edge is a unique technique for extracting exclusively Sc(III)–OH_2_ interactions, but it is inherently limited to the nearest-neighbor interactions around Sc(III). The results of MD simulations significantly depend on the force fields employed; the running coordination number of Sc(III) is obtained using Equation (5) (Section 4) as a mean *CN* of Sc(III). Thus, MD simulations alone sometimes fail to reproduce X-ray/neutron scattering data accurately. Our EPSR analysis, combined with neutron scattering data, reproduces the experimental neutron data and extracts the pdf of Sc(III)–Ow pairs exclusively.

The number of water molecules surrounding Sc(III) in aqueous solutions is influenced by the water network structure, which is altered by temperature and pressure. When the temperature is elevated and the pressure is applied, the tetrahedral network structure of water is distorted and/or broken down. This fact means that more free water molecules are generated and tend to surround Sc(III) as well as Cl^−^. This situation is also the case for the predominant formation of [Sc(OH_2_)_7_]^3+^ at 1 GPa/298 K. The substantially increased *CN* of Cl^−^ at the GPa pressure and 523 K is also the case. The coordination of Sc(III) is affected by coordinating anions, such as Cl^−^and NO_3_^−^. Since Sc(III) is a labile ion, i.e., a rapid substitution reaction between water molecules and coordinating anions (Cl^−^ and NO_3_^−^), the ion association between Sc(III) and Cl^−^ or NO_3_^−^ occurs. Since the electrostatic attraction, *F*, between two ions (the electric charge z_+_ and z_-_) separated by a distance *r* in a solvent such as relative permittivity *ε*_r_ (=*ε*/*ε*_0_, *ε* is the permittivity of a solvent) is expressed using Equation (1) [30].

(1) $$\left(F=\frac{z_{+}z_{-}e^{2}}{4\pi \varepsilon_{0}\varepsilon_{r}r^{2}}\right)$$

Here, *ε*_0_ is the vacuum permittivity, equal to 8.854 × 10^−12^ F m^−1^.

The electrostatic attraction between Sc(III) and Cl^−^ is proportional to the electric charge and inversely proportional to the square of the distance and the relative permittivity of water, i.e., the ion association between Sc(III) and Cl^−^ depends on the relative permittivity of water. The relative permittivity of bulk water (H_2_O) in the four thermodynamic states was calculated according to the literature [31] to be 78.6, 95.6, 38.3, and 64.3 at 0 GPa/298 K, 1 GPa/298 K, 1 GPa/523 K, and 4 GPa/523 K, respectively. The highest value at 1 GPa/298 K suggests the least amount of ion association, as evidenced by the predominant [Sc(OH_2_)_7_]^3+^ in Table 2. The lowest value at 1 GPa/523 K promotes the ion association of the triplets. In conclusion, Sc(III) takes the predominant [Sc(OH_2_)_7_]^3+^ in a pentagonal bipyramid with a Sc(III)–Ow distance of 2.11 Å, which coexists with [Sc(OH_2_)_6_]^3+^ and [Sc(OH_2_)_8_]^3+^ in an aqueous solution. Most of the methods employed so far provide an average feature of Sc(III) hydration, with individual structure information depending on the intrinsic principle and limitations of each method. A tendency of contact ion association between Sc(III) and Cl^−^ can be estimated from the dielectric constant of bulk water in the thermodynamic state. In the gigapascal pressure range, the tetrahedral network structure of water transforms into a densely random-packed structure, substantially enhancing the hydration number of a weakly solvated chloride ion, but does not affect the solvation of a strongly solvated Sc(III). The present outcomes will provide a key to understanding the underlying mechanisms of reactions and equilibria of metal complexes under high pressure, in terms of reaction and activation volumes in coordination chemistry, various processes in the upper mantle of the Earth in geology, and the pressure induced protein folding and unfolding phenomena in biology.

## 4. Materials and Methods

### 4.1. Sample Preparation

**Figure 8:**
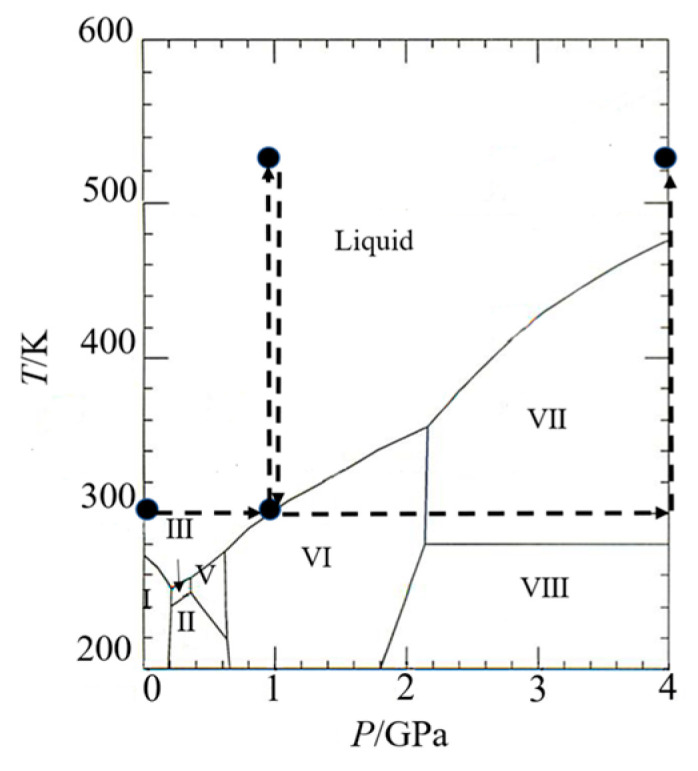
Phase diagram of water [34]. The solid circles show the thermodynamic conditions measured in this study. The dashed lines represent the order in which the experiments were performed. Symbols I to VIII show the phases of ice.

Scandium chloride (99.99%, Sigma-Aldrich, Tokyo, Japan), commercially available, was dried at 393 K for 4 h and cooled in a desiccator. A 1 mol dm^−3^ ScCl_3_ aqueous solution was prepared by dissolving dry ScCl_3_ into D_2_O (99.8 atom% D, Kanto Chemical Co., Inc., Tokyo, Japan). One molality as solute concentration was chosen to assure that the ion-related contribution was as significantly high as in the total pdf of the solution (see Supplementary Materials) and the formation of individual solvation shells of ions. Deuterated hydrochloric acid (DCl, 35 wt%, 99 atom% D, Sigma-Aldrich, Tokyo, Japan), commercially available, was added to the ScCl_3_ solution so that the pH became 2.2 to prevent the hydrolysis of Sc(III). All preparations were performed in a nitrogen-filled glove box to avoid contamination of light water. It should be noted that the incoherent scattering cross section of H is 40 times larger than that of D ($\sigma_{inc}^{H}=80.27\;barn,\;\sigma_{inc}^{D}=2.04\;barn)\;$ [32] causing the large incoherent scattering from a sample solution and an inelasticity effect. The density of a sample solution at 298 K was measured using a vibrational densitometer (DMA48, Anton Paar, Tokyo, Japan). Those in the GPa pressure range were calculated from the density of water at the corresponding thermodynamic states [33]. Table 5 summarizes the concentrations and densities of the sample solution at the measured temperatures and pressures (Figure 8).

### 4.2. Neutron Scattering Measurements

Neutron scattering experiments were performed using a high-pressure diffractometer PLANET [35] installed at beamline BL11 of a spallation neutron facility J-PARC MLF, Tokai, Japan. The 320 ^3^He position-sensitive detectors were arranged at a scattering angle (2*θ*) of 90° for both sides of the diffractometer, with horizontal and vertical coverages of 90° ± 11.3° and 0° ± 34.6°, respectively. The pulsed neutron wavelength range (*λ*) used was 0.2−12 Å. The amplitude of the wavevector *Q* (=4πsin*θ*/*λ*) accessible was 0.8–40 Å^−1^.

For measurements under ambient conditions (298 K, 0.1 MPa), a sample solution was placed into a cylindrical vanadium can with an inner diameter of 2.8 mm, a thickness of 0.1 mm, and a height of 30 mm, which was sealed with an indium wire. An empty can, an empty background, and a vanadium rod of the same dimensions as the can were measured. For high-pressure measurements at 1 GPa/298 K, 1 GPa/523 K, and 4 GPa/523 K (see Figure 1), a sample solution was transferred into a Teflon cell with an inner diameter of 5.5 mm and an inner height of 6.4 mm to prevent corrosion by an aqueous salt solution at high temperatures. The sample cell was inserted in a high-pressure assembly (Figure S1, Supplementary Materials) and compressed with a six-axis multi-anvil press ATSUHIME [36] to generate 4 GPa. A NaCl pellet was placed under the Teflon cell as a pressure marker. The sample pressure was estimated from the shift of the Bragg peaks of NaCl based on the calibration curve. A sample solution was heated by applying AC to a graphite heater surrounding the Teflon cell. The sample temperature was calculated using the relationship between the cell temperature and the AC power. The maximum pressure measured in this study was 4 GPa. The maximum temperature examined was limited to 523 K because Teflon degrades at high temperatures. A vanadium rod loaded in the cell with the same dimensions as the sample cell was separately measured at the same thermodynamic states as the sample measurements. An empty cell with the same dimensions as the sample cell at each thermodynamic state was also measured under ambient conditions. The sample intensity is normalized to an absolute unit (barn) using this data.

### 4.3. Neutron Data Treatment

Small Bragg peaks from a vanadium rod were removed, and the intensity in this region was interpolated using the data below and above this range. The total cross-sections of a sample, a vanadium rod, and an empty cell were normalized by the proton intensities and binned in increments of *Q* = 0.01 Å^−1^. Then, the scattered data were corrected for absorption by the sample and the cell and multiple scattering and then normalized to the absolute units by using the vanadium rod and empty-cell data, followed by subtraction of the incoherent scattering to give the structure factor *S*(*Q*) defined by Equation (2). All the data treatments were performed with a program nvaSq [37].

(2) $$\left(\left(Q\right)=\frac{\left(\frac{d\sigma }{d\Omega }\right)-\left\{(\sum x_{i}b_{i}^{2})-{(\sum x_{i}b_{i})}^{2}\right\}}{{(\sum x_{i}b_{i})}^{2}}\right)$$

where $\left(\frac{d\sigma }{d\Omega }\right)$ is the differential scattering cross-section, *x_i_* and *b_i_* are the atomic fractions, and the coherent scattering length of atom *i*, respectively. The coherent scattering length, the absorption, and the incoherent cross-sections of atoms were taken from the literature [32]. The absorption and incoherent cross-sections for D were calculated from a least-squares fitting procedure using the experimentally obtained total cross-section of D_2_O [38]. The Kameda method corrected the inelasticity effect [39]. The remaining unphysical baseline was further subtracted with a third-order polynomial function using the Origin Pro 2022 software [40].

### 4.4. EPSR Calculations

The structure factors *S*(*Q*) were transformed into the interference functions *F*(*Q*) used in the EPSR calculations by Equation (3). At this stage, an overall scaling factor of *F*(*Q*) was estimated by a least-squares fitting procedure in which the experimental *F*(*Q*) values were compared with the theoretical ones of the intramolecular O–D and D–D interactions in a D_2_O molecule in the high *Q*-region above 10 Å^−1^. An overall scaling factor, the intramolecular distances, and the root mean square amplitudes were optimized using OriginPro. The corresponding total pair distribution functions *G*(*r*) were obtained by the Fourier transform of the *F*(*Q*) by Equation (4). The spurious ripples observed in a *r*-region below 0.80 Å in *G*(*r*) were corrected in a usual manner [37].

(3) $$\left(F\left(Q\right)={[(\sum x_{i}b_{i})}^{2}(S\left(Q\right)-1)]\right)$$

(4) $$\;G\left(r\right)=1+\frac{1}{{2\pi }^{2}r\rho_{0}}\int_{Q_{min}}^{Q_{max}}QF(Q)\sin\left(Qr\right)dQ$$

EPSR calculations were performed in a unit cell to reproduce the composition of the sample solution, i.e., 20 Sc^3+^, 60 Cl^−^, and 1000 D_2_O. Monte Carlo calculations were performed using the pair potentials for Sc [18], Cl [41], and D and O [42] to equilibrate the system. Then, the pair potentials were empirically modified from the initial ones, followed by Monte Carlo calculations repeatedly until good agreements between the simulated and experimental *F*(*Q*) data were obtained. Once good agreements were obtained, we calculated the pair distribution functions (pdfs), the coordination number (*CN*) distributions, the angular distributions, and the spatial density functions (SDFs) to determine the 3D structure. The coordination number *CN* of atom *j* around atom *i* was calculated from the corresponding pair distribution function *g_ij_*(*r*) by Equation (5).

(5) $${\;CN}_{ij}=4\pi \rho_{j}\int_{r_{min}}^{r_{max}}g_{ij}\left(r\right)r^{2}dr$$

where *ρ_j_* was the number density of atom *j*, and *r_min_* and *r_max_* were the lower and upper limits of integration. In the present study, *r_max_* was the *r*-value at the first minimum of the corresponding pdf function. The parameters and references for each element in EPSR calculations are listed in Table 6. The details of EPSR calculations have been described elsewhere [43].

## Figures and Tables

**Table 1:** The Sc(III)-O distance (*r*) and coordination number (*CN*) obtained by various methods. Ow and O_N_ denote water oxygen and nitrate oxygen atoms, respectively. The number of parentheses in the interatomic distance is the estimated experimental uncertainty.

Method	*r*/Å	*CN*	System	Refs.
NS + EPSR	2.11(2) (Ow), 2.42(2) (Cl)	5.2 (Ow), 1.4 (Cl)	1 *m* ScCl_3_ in 0.01 *m* DCl at 0.1 MPa, 298 K	This work
	2.11(2) (Ow), 2.42(2) (Cl)	6.6 (Ow), 0.4 (Cl)	at 1 GPa, 298 K	
	2.11(2) (Ow), 2.42(2) (Cl)	5.0 (Ow), 1.4 (Cl)	at 1 GPa, 523 K	
	2.11(2) (Ow), 2.44(2) (Cl)	5.2 (Ow), 1.5 (Cl)	at 4 GPa, 523 K	
XS + EPSR + XANES + DV-Xα	2.14 (Ow), 2.14 (O_N_)	6.2 (Ow), 1.2 (O_N_)	0.96 M Sc(NO_3_)_3_ in 0.06 M HNO_3_	[18]
^1^H-NMR	n.a.	n.a.	Sc(ClO_4_)_3_, Sc(NO_3_)_3_ in water-acetone mixture	[7]
Raman	n.a.	7	Sc(NO_3_)_3_.20H_2_O	[8]
Raman	n.a.	7	2.2 M Sc(ClO_4_)_3_ at pH = 1.6	[9]
Raman + HF-MP2 MO	2.18	6	1.65 M Sc(ClO_4_)_3_ in 0.16 M HClO_4_	[10,11]
LAXS + EXAFS	2.18	7	1.06, 3.00 M Sc(ClO_4_)_3_ in 0.01 M HClO_4_	[12]
LAXS	2.15	7	1, 3 M Sc(ClO_4_)_3_ in 0.01 M HClO_4_	[13]
EXAFS + LAXS	2.17(1)(6×), 2.32(4), 2.5	8	0.98 M Sc(ClO_4_)_3_ in 0.88 M HClO_4_	[14]
EXAFS + QM/MD	2.16	8	0.2 M Sc(CF_3_SO_3_)_3_	[14]
MD	2.16	10	1 Sc(III) in 819 H_2_O	[14]
QM/MM/MD	2.14(6×), 2.26(1×)	7	1 Sc(III) in 499 H_2_O	[16]
QMCF MD	2.14	6.03	1 Sc(III) in 2000 H_2_O	[17]
RI-MP2 QM/MM/MD	2.15	6	1 Sc(III) in 2000 H_2_O	[17]

NS: neutron scattering, XS: X-ray scattering, EPSR: empirical potential structure refinement, XANES: X-ray absorption near-edge structure, DV-Xα: discrete variational Xα, MO: molecular orbital, EXAFS: extended X-ray absorption fine structure, QM/MD: quantum mechanics/molecular dynamics, QMCF: quantum mechanical charge field, RI-MP2: resolution-of-identity MP2, QM/MM/MD: quantum mechanics/molecular mechanics/molecular dynamics.

**Table 2:** Interatomic distance (*r*) and coordination numbers (*CN*) of each atom pair in a 1 *m* ScCl_3_ aqueous solution under the four thermodynamic conditions. *r*_max_ is the upper limit (the first minimum distance) of each pair distribution function used in Equation (4) in Section 4. I and II denote the first and second shells of Sc(III) and Cl^−^, respectively. The estimated experimental uncertainties are ±0.02 and ±0.04 Å for the first and second coordination shells, respectively.

Conditions	Parameters	Sc–Ow(I)	Sc–Hw(I)	Sc–Ow(II)	Cl–Ow(I)	Cl–Hw(I)	Cl–Ow(II)
0 GPa 298 K	*r*/Å	2.11	2.79	4.31	3.10	2.14	5.64
	*CN*	5.2 ± 1.6	10.4 ± 3.2		7.8 ± 1.4	4.7 ± 2.2	
	*r*_max_/Å	2.90	3.33		3.89	2.81	
1 GPa 298 K	*r*/Å	2.11	2.81	4.35	3.05	2.12	5.62
	*CN*	6.6 ± 1.2	13.5 ± 1.7		9.7 ± 1.2	6.8 ± 1.5	
	*r*_max_/Å	2.64	3.30		3.92	2.79	
1 GPa 523 K	*r*/Å	2.11	2.81	4.42	3.11	2.20	5.72
	*CN*	5.0 ± 1.6	10.2 ± 3.2		8.3 ± 1.7	4.2 ± 2.1	
	*r*_max_/Å	2.64	3.30		3.92	2.79	
4 GPa 523 K	*r*/Å	2.11	2.78	4.45	3.10	2.17	5.78
	*CN*	5.2 ± 1.8	10.3 ± 3.5		10.9 ± 1.7	4.6 ± 2.2	
	*r*_max_/Å	2.96	3.30		4.18	2.76	

**Table 3:** Interatomic distance (*r*) and coordination numbers (*CN*) of Sc(III) association with chloride ion in a 1 *m* ScCl_3_ aqueous solution under the four thermodynamic conditions. *r*_max_ is the upper limit (the first minimum distance) of each pair distribution function used in Equation (4). I and II denote the first and second shells of Sc(III), respectively. The estimated experimental uncertainties are ±0.02 and ±0.04 Å for the first and second coordination shells, respectively.

Abbreviation	Parameters	Sc–Cl(I)	Sc–Cl(II)	Sc–Sc
0 GPa 298 K	*r*/Å	2.42	4.44	4.88
	*CN*	1.4 ± 1.1		0.056 ± 0.24
	*r*_max_/Å	3.04		5.32
1 GPa 298 K	*r*/Å	2.42	4.44	n.a.
	*CN*	0.38 ± 0.80		
	*r*_max_/Å	3.00		
1 GPa 523 K	*r*/Å	2.42	4.53	4.86
	*CN*	1.4 ± 1.1		0.43 ± 0.63
	*r*_max_/Å	3.00		5.38
4 GPa 523 K	*r*/Å	2.44	4.53	4.89
	*CN*	1.5 ± 1.2		0.22 ± 0.42
	*r*_max_/Å	3.05		5.30

**Table 4:** Interatomic distance (*r*) and coordination numbers (*CN*) of solvent water in a 1 *m* ScCl_3_ aqueous solution under the four thermodynamic conditions. *r*_max_ is the upper limit (the first minimum distance) of each pair distribution function. I, II, and III denote the first, second, and third shells of a water molecule, respectively. The estimated experimental uncertainties are ±0.02, ±0.04, and ±0.06 Å for the first, second, and third coordination shells, respectively.

Abbreviation	Parameters	Ow-Ow(I)	Ow-Hw(I)	Ow-Ow(II)	Ow–Hw(II)	Ow-Ow(III)	Ow–Hw(III)
0 GPa 298 K	*r*/Å	2.77	1.83	4.13	3.28	7.12	6.90
	*CN*	5.5 ± 1.5	2.1 ± 1.1				
	*r*_max_/Å	3.52	2.31				
1 GPa 298 K	*r*/Å	2.78	1.86	5.94	3.30	8.97	6.21
	*CN*	14.3 ± 1.6	1.94 ± 1.1				
	*r*_max_/Å	4.50	2.28				
1 GPa 523 K	*r*/Å	2.84	1.95	5.55	3.20	8.24	5.74
	*CN*	11.9 ± 2.0	1.9 ± 1.1				
	*r*_max_/Å	4.50	2.28				
4 GPa 523 K	*r*/Å	2.89	2.08	5.68	3.20	8.33	5.78
	*CN*	12.6 ± 1.8	1.8 ± 1.1				
	*r*_max_/Å	4.37	2.18				

**Table 5:** The concentration (*c*) and density (*d*) of a ScCl_3_ aqueous solution in D_2_O and the number density (*ρ*_S_ and *ρ*_V_) of the sample solution and a vanadium rod, respectively, in different thermodynamic states.

Abbreviation	*c*/mol dm^−3^	*d*/g cm^−3^	*ρ*_S_/atoms Å^−3^	*ρ*_V_/atoms Å^−3^	*p*/GPa	*T*/K
0 GPa 298 K	1.113	1.247	0.1009	0.07258	1.0 × 10^−4^	298
1 GPa 298 K	1.320	1.479	0.1197	0.07301	0.66	298
1 GPa 523 K	1.140	1.277	0.1034	0.07238	0.49	523
4 GPa 523 K	1.285	1.440	0.1165	0.07324	3.54	523

**Table 6:** Potential parameters for each element used in EPSR calculations of a 1 mol/kg ScCl_3_ aqueous solution in D_2_O.

Atoms	*ε*/kJ mol^−1^	*σ*/Å	Atomic Mass	Coulomb Charge/(e)
O_W_	0.650	3.165	16.0	−0.8480
H_W_	0.00	0.00	2.0	0.4240
Cl^−^	0.710	4.02	35.452	−1
Sc^3+^	26.3	1.50	44.956	3

## Data Availability

All data in the manuscript are available on request.

## Supplementary Materials

The following supporting information can be downloaded at https://www.mdpi.com/article/10.3390/molecules30163417/s1, Figure S1: Interference functions of a 1 *m* ScCl_3_ aqueous solution in D_2_O under the different thermodynamic states. Table S1. Intramolecular parameters of a D_2_O molecule under the four different thermodynamic states determined by the least-squares fitting procefure, together with those in polymorphs ice for comparison, Figure S2: High-pressure cell assembly used in the neutron scattering experiments [25,26,27,28].
