# Supplementary material for: Scandium(III) Solvation and Association and Water Structure in the Gigapascal Pressure Range Investigated by Neutron Scattering
Source: Molecules. 2025 Aug 19;30(16):3417. doi: 10.3390/molecules30163417 (PMC12388061; doi:10.3390/molecules30163417)
Supplement: Supplementary file 1 [file molecules-30-03417-s001.zip › molecules-3777258-supplementary.pdf]

Supplemental Materials

# Scandium(III) Solvation and Association and Water Structure in the Gigapascal Pressure Range Investigated by Neutron Scattering

**Toshio Yamaguchi [1,2,*], Sinichi Machida [3] and Takanori Hattori [4]**

[1] Key Laboratory of Comprehensive and Highly Efficient Utilization of Salt Lake Resources, Key Laboratory of Salt Lake Resources Chemistry of Qinghai Province, Qinghai Institute of Salt Lakes, Chinese Academy of Sciences, Xining 810008, China
[2] Department of Chemistry, Faculty of Science, Fukuoka University, Jonan, Fukuoka 814-0180, Japan
[3] Neutron Science and Technology Center, Comprehensive Research Organization for Science and Society, Tokai, Naka 319-1106, Japan; s_machida@cross.or.jp
[4] J-PARC Center, Japan Atomic Energy Agency, Tokai, Naka 319-1195, Japan; takanori@post.j-parc.jp
* Correspondence: yamaguch@fukuoka-u.ac.jp

## 1. Determination of the intramolecular structure of a water molecule

The interference functions over a $Q$-range of 10 to 40 Å$^{-1}$ are precominantly contributed by the intramolecular structue of a water molecule. In the least-squares fitting procedure, the function $U$ is minimized with independent variables, the interatomic distances ($r_{ij}$) and root-mean-squares displacements ($l_{ij}$) of an atom pair i-j, which are allowed to vary over a $Q$-range from $Q_{min}$ to $Q_{max}$.

$$U = \sum_{Q_{min}}^{Q_{max}} \{F(Q)_{obs} - F(Q)_{cal}\}^2, \qquad (1)$$

$$F(Q)_{cal} = x_O \left\{4b_O b_D \exp\left(-\frac{l_{OD}^2 Q^2}{2}\right) \sin(Qr_{OD}) /(Qr_{OD}) + 2b_D^2 (\exp\left(-\frac{l_{DD}^2 Q^2}{2}\right) \sin(Qr_{DD}) /(Qr_{DD})\right\}. \qquad (2)$$

where $x_O$ represens the atomic fraction of the water oxygen atom, and $b_i$ denotes the coherent scattering length of atom i. Figure S1 shows comparison between the experimental and fitted values and the residual funccions, $\Delta(Q)$, under the four different thermodynamic states. The optimized parameter values are given in Table S1

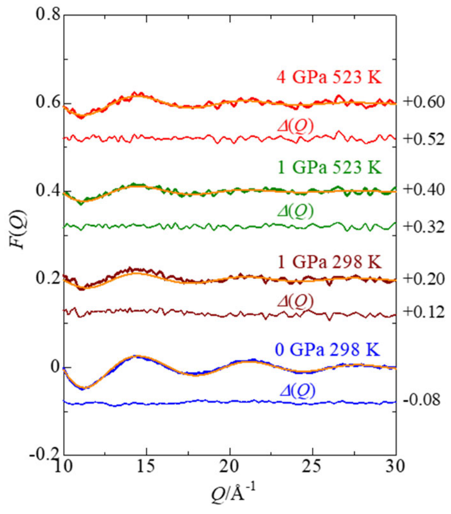


**Figure S1**. Interference functions of a 1 *m* $ScCl_3$ aqueous solution in $D_2O$ under the different thermodynamic states. Large dots and solid lines and small dot are the experimental, fitted values, and the residual functions, $\Delta(Q)$, respectively. Redidual values between the expare shown below.

**Table S1**. Intramolecular parameters of a $D_2O$ molecule under the four different thermodynamic states determined by the least-squares fitting procefure, together with those in polymorphs ice for comparison.

| Parameters | 0 GPa/298 K | 1 GPa/298 K | 1 GPa/523 K | 4 GPa/523 K | Liquid $D_2O$ | Ice Ih | Ice Ic | Ice VI | Ice VIII |
|---|---|---|---|---|---|---|---|---|---|
| $r_{OD}$/Å | 0.9594 (3) | 0.9693 (15) | 0.9626 (3) | 0.9697 (11) | 0.974 (1) | 1.006 (4) | 1.01 | 0.961 (94) | 0.9685 (71) |
| $l_{OD}$/Å | 0.0559 (6) | 0.0510 (31) | 0.0836 (21) | 0.0748 (20) | 0.064 (1) | | | | |
| $r_{DD}$/ Å | 1.546 (3) | 1.434 (15) | 1.535 (19) | 1.552 (68) | 1.53 (2) | 1.643 | 1.597 | 1.569 | 1.543 |
| $l_{DD}$/Å | 0.127 (2) | 0.120 (10) | 0.177 (15) | 0.229 (51) | 0.11 (1) | | | | |
| ∠D–O–D/° | 107.4 | 95.5 | 105.8 | 106.3 | 103.5 | 109.5 | 104.5 | 109.4 | 105.6 |
| $R^2$-value* | 0.9574 | 0.6059 | 0.7863 | 0.7968 | Ref. [1] | Ref. [2] | Ref. [3] | Ref. [4] | Ref. [4] |

*$R^2$ is the coefficient of determination.

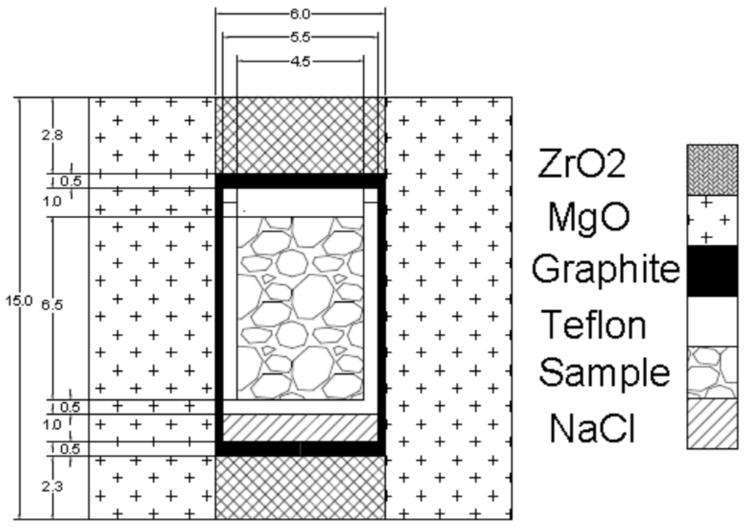


**Figure S2.** High-pressure cell assembly used in the neutron scattering experiments.

## References


1. Kameda, Y.; Amo, Y.; Usuki, T.; Umebayashi, Y.; Watanabe, H.; Ikeda, K.; Otomo, T. Direct determination of intramolecular structure of $D_2O$ in the first hydration shell of $Ni^{2+}$, *J. Mol. Liq*. **2023**, *382*, 121927.
2. Kuhs, W.F.; Lehmann, M.S. The structure of the ice Ih by neutron diffraction, *J. Phys. Chem*. **1983**, *87*, 4312-4313.
3. Arnold, G.P.; Finch, E.D.; Rabideau, S.W.; Wenzel, R.G. Neutron diffraction study of ice polymorphs. III. Ice Ic, *J. Chem. Phys*. **1968**, *49*, 4365–4369.
4. Kuhs, W.F.; Finney, J.L.; Vettier, C.; Bliss, D.V. Strucure and hydrogen ordering in ices VI, VII, and VIII by neutron powder diffraction, *J. Chem. Phys*. **1984**, *81*, 3612–3623.
